# The Los Angeles-B esophagitis is a conclusive diagnostic evidence for gastroesophageal reflux disease: the validation of Lyon Consensus 2.0

**DOI:** 10.1093/gastro/goaf004

**Published:** 2025-03-12

**Authors:** Jing Chen, Peiwen Dong, Songfeng Chen, Qianjun Zhuang, Mengyu Zhang, Kaidi Sun, Feng Tang, Qiong Wang, Yinglian Xiao

**Affiliations:** Department of Gastroenterology, The First Affiliated Hospital of Sun Yat-sen University, Guangzhou, Guangdong, P. R. China; Department of Gastroenterology, The Third People’s Hospital of Chengdu, The Affiliated Hospital of Southwest Jiaotong University, Chengdu, Sichuan, P. R. China; Department of Gastroenterology, The First Affiliated Hospital of Sun Yat-sen University, Guangzhou, Guangdong, P. R. China; Department of Gastroenterology, The First Affiliated Hospital of Sun Yat-sen University, Guangzhou, Guangdong, P. R. China; Department of Gastroenterology, The First Affiliated Hospital of Sun Yat-sen University, Guangzhou, Guangdong, P. R. China; Department of Gastroenterology, The Third People’s Hospital of Chengdu, The Affiliated Hospital of Southwest Jiaotong University, Chengdu, Sichuan, P. R. China; Department of Gastroenterology, The Third People’s Hospital of Chengdu, The Affiliated Hospital of Southwest Jiaotong University, Chengdu, Sichuan, P. R. China; Department of Gastroenterology, The Third People’s Hospital of Chengdu, The Affiliated Hospital of Southwest Jiaotong University, Chengdu, Sichuan, P. R. China; Department of Gastroenterology, The First Affiliated Hospital of Sun Yat-sen University, Guangzhou, Guangdong, P. R. China

**Keywords:** Lyon Consensus 2.0, esophagitis, Los Angeles classification, acid-suppressive therapy, diagnosis

## Abstract

**Background and Aims:**

Recently, Lyon Consensus 2.0 recommended Los Angeles (LA)-B esophagitis as conclusive evidence and LA-A esophagitis as borderline evidence for gastroesophageal reflux disease (GERD). This study aimed to investigate the diagnostic value of LA-B and LA-A esophagitis.

**Methods:**

Patients with typical reflux symptoms who underwent endoscopy examination and received acid-suppressive therapy from two tertiary hospitals [the First Affiliated Hospital of Sun Yat-sen University (Guangzhou, P. R. China) and the Third People’s Hospital of Chengdu (Chengdu, P. R. China)] were retrospectively included. Acid-suppression response rates, endoscopy results, motility, and reflux parameters were compared between patients with different grades of esophagitis.

**Results:**

In total, 401 patients were enrolled, among whom 254 were without reflux esophagitis (RE), 51 had LA-A esophagitis, 44 had LA-B esophagitis, and 52 had LA-C/D esophagitis. Patients with LA-B esophagitis and LA-C/D esophagitis had significantly higher acid-suppressive response rates than non-RE patients (*P *<* *0.05), whereas no significant difference was found between patients with LA-A esophagitis and non-RE patients (non-RE vs LA-A vs LA-B vs LA-C/D: 52.4% vs 70.6% vs 75.0% vs 82.7%). Among patients with LA-A esophagitis, those with a number of reflux episodes that exceeded 80 per day (90.0% vs 52.4%, *P *=* *0.044) or hypotensive esophagogastric junction (72.4% vs 52.4%, *P *=* *0.040) had significantly higher acid-suppressive response rates than non-RE patients.

**Conclusions:**

LA-B esophagitis can be regarded as conclusive evidence for GERD and initiate acid-suppressive therapy. LA-A esophagitis did not establish a definite GERD diagnosis alone. When combined with adjunctive or supportive evidence, the acid-suppressive therapy response rate of LA-A esophagitis improved.

## Introduction

Gastroesophageal reflux disease (GERD) is a condition characterized by the reflux of gastric and/or duodenal contents into the esophagus, causing disturbing symptoms and/or complications [1]. GERD is not only one of the most common digestive tract diseases, affecting ∼8%–33% of the worldwide population [2], but also one of the diseases causing the most serious economic burden to both patients and society [3].

Currently, there are several methods that can be used to diagnose GERD [4, 5]. Symptomatology and proton-pump inhibitor (PPI)/potassium-competitive acid blocker (P-CAB) tests are commonly used in clinical practice. However, symptoms of GERD patients are highly heterogeneous and none of these symptoms is unique to GERD. As a result, the sensitivity and the specificity of GERD diagnosis by using typical reflux symptoms is only 38% and 89% [5, 6]. As for the PPI/P-CAB test, due to the placebo effect, although its sensitivity can reach 78%, its specificity is only 54% [5, 7]. In order to obtain a more conclusive diagnosis of GERD, patients still need to undergo objective examinations such as endoscopy and reflux monitoring. Compared with reflux monitoring, endoscopy is less costly and more widespread. Furthermore, endoscopy can help rule out digestive tract tumor diseases [8]. Therefore, it has long been regarded as an indispensable part of the diagnostic process for GERD.

Based on endoscopic manifestations, GERD can be classified into non-erosive reflux disease, reflux esophagitis (RE), and Barrett's esophagus. According to the Los Angeles (LA) classification, RE can be further divided into four grades based on the severity of esophageal mucosal damage, with LA-A being the mildest and LA-D being the most severe [9]. There is a strong positive correlation between the RE grades and the reflux severity [9]. Therefore, for a considerable period, the medical community held that high-grade esophagitis (LA-C and LA-D esophagitis) was conclusive evidence for GERD diagnosis. However, high-grade esophagitis accounts for only a small proportion of RE, especially in Asian countries (accounts for <5% of RE) [10]. For more clinically common RE types, LA-A- and LA-B-grade esophagitis can be found in ∼5.0%–7.5% of asymptomatic controls [11–13] but whether they can be used to establish GERD diagnosis alone has long been controversial.

Mainstream Western countries are opposed to regarding LA-A and LA-B esophagitis as conclusive diagnostic evidence of GERD [4, 14], whereas Asian countries tended to consider LA-B esophagitis as adequate to establish a definite diagnosis of GERD [5, 15]. The divergence between Asian and Western consensus on the above issue stems mainly from the lower reflux burden of Asian countries [10]. Recently, Lyon Consensus 2.0 updated the diagnosis criteria for GERD based on two perspective studies in the West [16–18]. Remarkably, this is the first global consensus that clearly pointed out that LA-B esophagitis should be regarded as conclusive diagnostic evidence for GERD. This redefinition will significantly increase the value of endoscopy in the diagnosis of GERD. However, this also leads to another concern: Could it lead to overdiagnosis of GERD?

As far as we know, there are limited studies of the reliability of using LA-B esophagitis to diagnose GERD. The objective of this study is to evaluate the diagnostic value of LA-B esophagitis for GERD and to explore whether adjunctive and supportive diagnostic evidence can further improve the diagnostic efficacy of LA-A esophagitis for GERD.

## Methods

### Population

Consecutive adult outpatients (18–80 years old) who presented with predominant heartburn or reflux symptoms (occurring at least 2 days/week and lasting for ≥3 months) and received endoscopy examination and completed 8 weeks of PPI or 4 weeks of P-CAB therapy were retrospectively enrolled from two tertiary hospitals [the First Affiliated Hospital of Sun Yat-sen University (Guangzhou, P. R. China) and the Third People’s Hospital of Chengdu (Chengdu, P. R. China)] from 2019 to 2023. Patients with upper digestive tract surgery history, gastrointestinal cancer, or major motility disorders were excluded. The study was approved by the Ethical Review Board of Sun Yat-sen University (No. [2021] 275).

### Endoscopy examination

Endoscopy examination was performed in the left-lateral decubitus position by experienced endoscopists. Hiatus hernia was considered if the level of the rise of the endoscopically visible rugal folds dislocated ≥1.0 cm above the level of the diaphragmatic impression [19]. According to Lyon Consensus 2.0, hiatus hernia under endoscopy examination was regarded as adjunctive or supportive evidence of GERD [18]. The presence and severity of esophagitis were evaluated based on the LA classification, which is as follows [9]: (i) LA-A represents one or more erosion(s) of <5 mm that do not extend between the tops of two mucosal folds; (ii) LA-B corresponds to one or more erosion(s) of >5 mm long that do not extend between the tops of two mucosal folds; (iii) LA-C is one or more erosion(s) that are continuous between the tops of two or more mucosal folds that involve <75% of the circumference; (iv) LA-D is one or more erosion(s) that involve at ≥75% of the oesophageal circumference.

### High-resolution manometry

The procedure was conducted as previously reported [20]. In brief, the high-resolution manometry (HRM) examination process included a 30-second basal pressure recording period, with ten  5-mL liquid swallows in the supine position, and another 30-second basal pressure recording period, with five 5-mL liquid swallows in the upright position. Lower esophageal sphincter (LES) basal pressure, LES length, esophagogastric junction (EGJ) contractile integral, EGJ inspiratory pressure, EGJ expiratory pressure, integrated relaxation pressure, distal contractile integral, distal latency, EGJ morphology, and manometric diagnosis were measured based on the Chicago classification version 4.0 [21]. According to Lyon Consensus 2.0, hypotensive EGJ, hiatus hernia, and ineffective esophageal motility absent contractility were regarded as adjunctive or supportive evidence of GERD [18].

### Multichannel intraluminal impedance-pH monitoring

Multichannel intraluminal impedance-pH (MII-pH) monitoring was performed after discontinuation of PPI/potassium-competitive acid blocker for ≥7 days, antacid for ≥1 day, and prokinetics and histamine H2 antagonists for ≥3 days [22]. The specific procedure was conducted as previously reported [20]. Meal periods were excluded during the analysis. Acid exposure time (AET), mean nocturnal baseline impedance (MNBI), post-reflux swallow-induced peristaltic wave index, proximal reflux events, total reflux events, and symptom association probability were calculated and recorded [22]. Positive symptom association probability, total reflux episodes numbering >80/day and MNBI of <1,500Ω were considered to be adjunctive or supportive evidence of GERD [18].

### Symptom evaluation and outcome definition

The frequency and severity of the predominant symptom were assessed on a four-point Likert scale (frequency: 0, none; 1, 1 day/week; 2, 2–3 days/week; and 3, 4–7 days/week; severity: 0, none; 1, mild; 2, moderate; and 3, severe) both at the time the patients were enrolled and at the end of their treatments. A composite symptom score was obtained by multiplying the frequency score by the severity score. A response to acid-suppressive therapy was considered positive if the composite symptom score during the final week of PPI/P-CAB treatment decreased by >50% from baseline [23]. A gastroesophageal reflux disease questionnaire was also used to evaluate the patients’ symptom situations.

### Statistical analysis

Analyses of the endoscopy, HRM, and reflux monitoring were all conducted by two independent experienced investigators. All statistical analyses were conducted by using SPSS 26 (IBM, Armonk, NY, USA). Mean ± standard deviation was used for continuous variables with normal distribution and median (interquartile range) for those without normal distribution. Categorical variables were presented as percentages. Univariate analyses were performed by using parametric (Student’s *t*-test and one-way ANOVA) or non-parametric (Mann–Whitney *U* test and Kruskal–Wallis tests) methods for continuous variables and the chi-square test was used for categorical variables. The *P*-values are two-sided and considered significant when <0.05.

## Results

In total, 401 patients (54.6% males, median age 47 years) were enrolled in this study, among whom 254 (63.3%) were non-RE patients, 51 (12.7%) had LA-A esophagitis, 44 (11.0%) had LA-B esophagitis, and 52 (13.0%) had LA-C/D esophagitis. The basal characteristics are listed in Table 1. No significant difference was found in terms of age, gastroesophageal reflux disease questionnaire score, and the composite symptom score. Patients with LA-B or LA-C/D esophagitis had a higher proportion of male gender and hiatus hernia under endoscopy examination than patients without esophagitis. RE patients all had a higher body mass index than non-RE patients.

**Table 1.** Baseline information for patients with different grades of esophagitis

Characteristic	Non-RE (*n *=* *254)	LA-A (*n *=* *51)	LA-B (*n *=* *44)	LA-C/D (*n *=* *52)	*P*-value
Male	115 (45.3)^a^	30 (58.8)^a,b^	34 (77.3)^b^	40 (76.9)^b^	<0.001
Age, years	46.0 (33.0, 58.0)	44.0 (33.0, 57.0)	46.5 (37.5, 55.0)	54.5 (36.5, 63.0)	0.135
Body mass index, kg/m^2^	22.0 ± 3.4	24.0 ± 4.0^c^	23.8 ± 3.8^c^	24.5 ± 2.6^c^	<0.001
Gastroesophageal reflux disease questionnaire	9.0 (7.0, 11.0)	9.0 (8.0, 11.0)	9.0 (7.8, 11.0)	9.0 (8.0, 10.0)	0.671
Composite symptom score	6.0 (4.0, 6.0)	6.0 (4.0, 6.0)	6.0 (4.0, 9.0)	6.0 (4.0, 9.0)	0.114
Hiatus hernia on endoscopy	27 (10.6)^d^	7 (13.7)^d,e^	12 (27.3)^e,f^	20 (38.5)^f^	<0.001

Data are presented as mean ± standard deviation or median (interquartile range) or number (percentage).

a–f There was no significant difference among groups with the same letter.

RE = reflux esophagitis, LA = Los Angeles.

**Figure 1.**
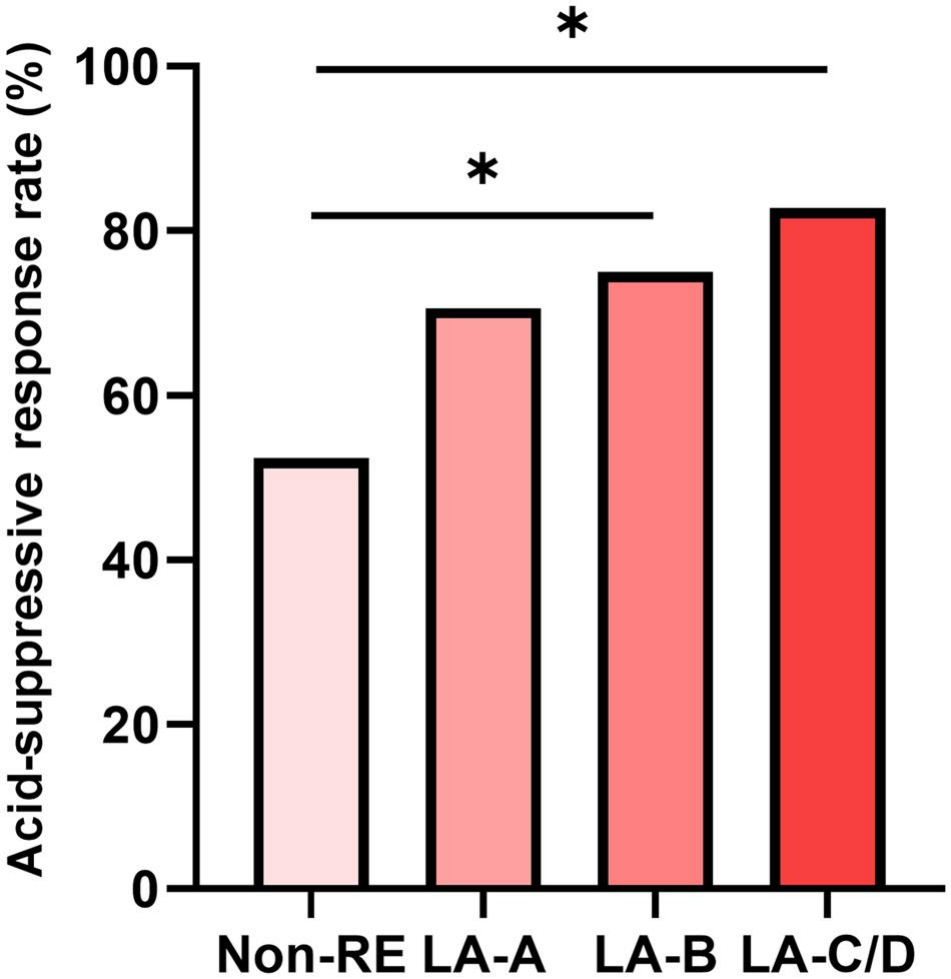
Acid-suppression response for patients with different grades of esophagitis. *P <* *0.05. Non-RE = non-reflux esophagitis, LA-A = Los Angeles-A reflux esophagitis, LA-B = Los Angeles-B reflux esophagitis, LA-C/D = Los Angeles-C/D reflux esophagitis.

Notably, the acid-suppressive response rate was significantly higher in patients with LA-B esophagitis and LA-C/D esophagitis compared with non-RE patients (*P *<* *0.05). However, this difference was not observed between patients with LA-A esophagitis and non-RE patients (non-RE vs LA-A vs LA-B vs LA-C/D: 52.4% vs 70.6% vs 75.0% vs 82.7%) (Figure 1).

### Esophageal motility profile among GERD patients

A total of 353 patients undertook HRM and MII-pH examination. The comparison of characteristics of esophageal motility between patients with different grades of esophagitis are listed in Table 2. Patients with LA-B exhibited lower EGJ inspiratory/expiratory pressure compared with non-RE patients. Patients with LA-C/D esophagitis also seemed to have lower EGJ inspiratory/expiratory pressure compared with non-RE patients, though this difference did not reach statistical significance due to the small sample size of patients with LA-C/D esophagitis. Meanwhile, patients with LA-B esophagitis had a worse motility status, as indicated by the lower distal contractile integral, and a higher rate of ineffective esophageal motility than non-RE patients. Patients with LA-C/D and LA-A esophagitis had a higher proportion of hiatus hernia under HRM examination than non-RE patients.

**Table 2.** Comparison of esophageal motility characteristics among patients with different grades of esophagitis

Parameter	Non-RE (*n *=* *253)	LA-A (*n *=* *51)	LA-B (*n *=* *40)	LA-C/D (*n *=* *9)	*P*-value
EGJ parameters					
EGJ inspiratory pressure, mmHg	19.0 (13.3, 26.9)^a^	17.8 (9.7, 25.3)^a,b^	13.0 (9.1, 20.9)^b^	13.9 (9.0, 19.4)^a,b^	0.002
EGJ expiratory pressure, mmHg	11.6 (7.1, 18.4)^c^	10.0 (6.2, 17.2)^c,d^	8.0 (4.0, 12.0)^d^	8.9 (4.4, 11.5)^c,d^	0.002
EGJ-CI, mmHg·cm	18.0 (4.1, 36.0)	19.4 (7.9, 43.5)	9.8 (0.5, 27.6)	12.4 (0.0, 37.0)	0.194
Integrated relaxation pressure, mmHg	6.7 (3.8, 9.8)	8.2 (5.0, 10.6)	6.2 (3.7, 8.7)	4.5 (0.7, 8.0)	0.069
EGJ morphology					<0.001
Type I	222 (87.7)^e^	32 (62.7)^f^	32 (80.0)^e,f^	4 (44.4)^f^	
Type II	25 (9.9)	14 (27.5)	6 (15.0)	2 (22.2)	
Type III	6 (2.4)	5 (9.8)	2 (5.0)	3 (33.3)	
Peristalsis parameters					
Distal latency, s	6.5 (5.7, 7.6)	6.6 (5.8, 7.4)	6.7 (6.2, 7.3)	7.0 (5.6, 7.8)	0.683
Distal contractile integral, mmHg·s·cm	760.2 (352.8, 1286.1)^g^	572.9 (307.7, 1186.8)^g,h^	456.2 (195.8, 940.2)^h^	486.6 (257.3, 818.8)^g,h^	0.005
Chicago classification					0.004
Normal	229 (90.5)	40 (78.4)	29 (72.5)	7 (77.8)	
Ineffective esophageal motility	24 (9.5)^i^	11 (21.6)^i,j^	11 (27.5)^j^	2 (22.2)^i,j^	
Hiatus hernia on high-resolution manometry, %	31 (12.3)^k^	19 (37.3)^l^	8 (20.0)^k,l^	4 (44.4)^l^	<0.001

Data were presented as median (interquartile range) or number (percentage).

a–l There was no significant difference among the groups with the same letter; RE = reflux esophagitis, LA = Los Angeles, EGJ = esophagogastric junction, CI = contraction integral.

### Reflux burden of GERD patients

The comparison results of MII-pH parameters are listed in Table 3. AET, the proportion of patients with pathological AET (>6.0%), and the DeMeester score increased with the severity of the esophagitis. Patients with esophagitis all had a higher AET, a higher proportion of patients with pathological AET, and a higher DeMeester score than those without. Patients with LA-B esophagitis had a higher number of acid reflux events than non-RE patients. Patients with LA-B or LA-C/D esophagitis had a lower MNBI, a lower proportion of MNBI >2,500 Ω, and a higher proportion of MNBI <1,500 Ω than non-RE patients.

**Table 3.** Comparison of MII-pH parameters among patients with different grades of esophagitis

Parameter	Non-RE (*n *=* *253)	LA-A (*n *=* *51)	LA-B (*n *=* *40)	LA-C/D (*n *=* *9)	*P*-value
Acid exposure					
AET, %	1.4 (0.4, 4.7)	3.0 (0.8, 11.5)^a^	6.7 (2.2, 13.9)^a^	9.7 (3.1, 17.9)^a^	<0.001
AET >6%	45 (17.8)	18 (35.3)^b^	23 (57.5)^b^	6 (66.7)^b^	<0.001
DeMeester score	5.6 (2.3, 16.4)	12.0 (4.1, 32.7)^c^	23.4 (8.0, 42.7)^c^	37.4 (12.7, 76.2)^c^	<0.001
Reflux episode					
Number of acid reflux events	24.0 (9.0, 37.0)^d^	32.0 (15.5, 43.8)^d,e^	38.0 (26.0, 56.0)^e^	25.5 (5.5, 31.8)^d,e^	0.001
Number of weakly acidic reflux events	19.0 (8.3, 31.8)^f^	13.0 (7.0, 23.0)^f,g^	12.0 (7.0, 18.0)^f,g^	5.5 (2.3, 12.5)^g^	0.005
Number of weakly alkaline reflux events	0.0 (0.0, 1.0)	0.0 (0.0, 1.0)	0.0 (0.0, 1.0)	0.0 (0.0, 0.0)	0.754
Number of total reflux events	47.5 (31.0, 68.0)	48.5 (29.3, 72.3)	55.0 (40.5, 84.5)	29.0 (16.5, 112.0)	0.244
Number of total reflux events >80	46 (19.2)^n^	10 (20.0)^r^	10 (27.0)^u^	3 (33.3)^x^	0.250
Baseline impedance					
MNBI, Ω	2,450.0 (1,057.4, 3558.2)^h^	2,250.0 (759.3, 3250.0)^h,i^	917.0 (540.0, 2200.0)^i^	641.0 (307.5, 1041.3)^i^	<0.001
MNBI >2,500 Ω	106 (48.6)^j^^,^^o^	18 (41.9)^j,k^^,^^s^	7 (20.0)^k^^,^^v^	1 (12.5)^k^^,^^y^	0.005
MNBI <1,500 Ω	75 (34.6)^l^^,^^p^	16 (37.2)^l,m^^,^^s^	23 (65.7)^m^^,^^v^	7 (87.5)^m^^,^^y^	<0.001
Others					
PSPWI, %	31.3 (19.0, 43.9)	26.1 (18.6, 38.8)	29.7 (12.4, 39.6)	25.0 (10.0, 28.5)	0.327
SAP	62 (28.8)^q^	16 (40.0)^t^	12 (41.4)^w^	3 (60.0)^z^	0.139

Data are presented as median (interquartile range) or number (percentage).

a–m There was no significant difference among the groups with the same letter (*P *>* *0.05).

n Total number of patients in the non-RE group was 240.

o Total number of patients in the non-RE group was 218.

p Total number of patients in the non-RE group was 217.

q Total number of patients in the non-RE group was 215.

r Total number of patients in the LA-A group was 50.

s Total number of patients in the LA-A group was 43.

t Total number of patients in the LA-A group was 40.

u Total number of patients in the LA-B group was 37.

v Total number of patients in the LA-B group was 35.

w Total number of patients in the LA-B group was 29.

x Total number of patients in the LA-C/D group was 9.

y Total number of patients in the LA-C/D group was 8.

z Total number of patients in the LA-C/D group was 5.

MII-pH = multichannel intraluminal impedance-pH, AET = acid exposure time, MNBI = mean nocturnal baseline impedance, PSPWI = post-reflux swallow-induced peristaltic wave index, SAP = symptom association probability.

### Verification of adjunctive or supportive evidence proposed in Lyon Consensus 2.0

**Figure 2.**
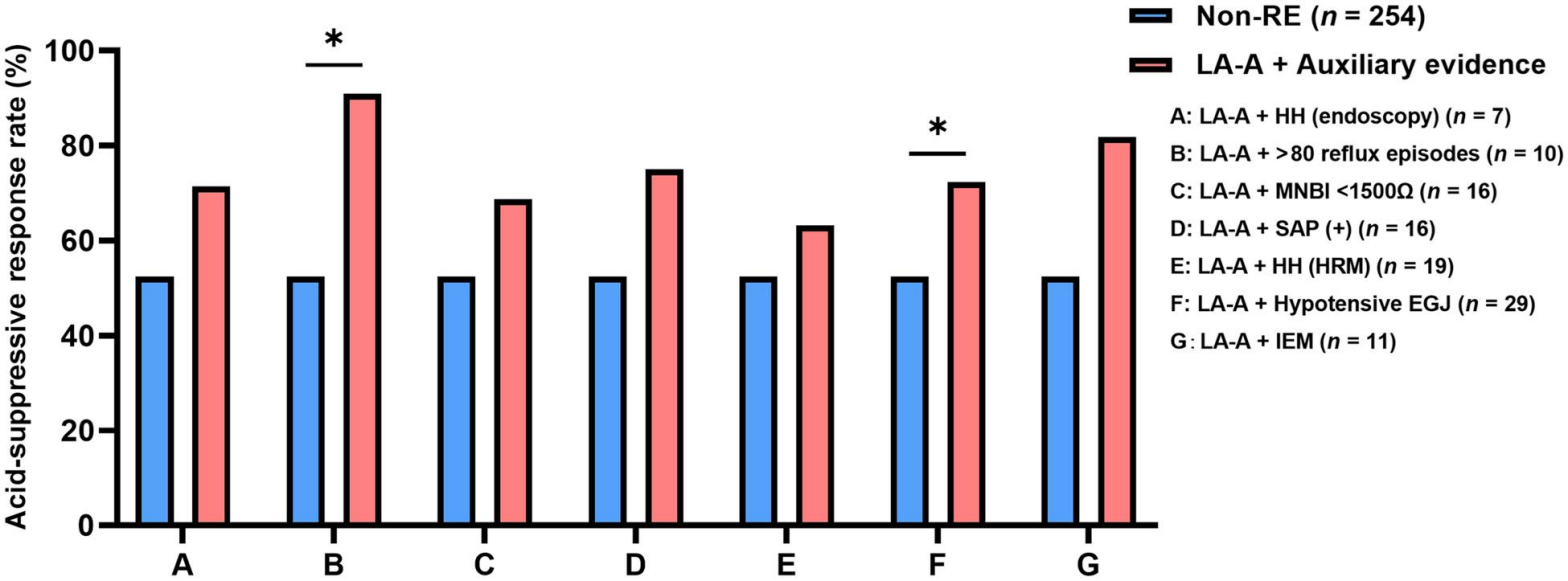
Acid-suppression response for patients with LA-A combined with auxiliary evidence and non-RE patients. *P <* *0.05. Non-RE = non-reflux esophagitis, LA-A = Los Angeles-A reflux esophagitis, HH = hiatal hernia, MNBI = mean nocturnal baseline impedance, SAP = symptom association probability, HRM = high-resolution manometry, EGJ = esophagogastric junction, IEM = ineffective esophageal motility.

The above results indicated that LA-B esophagitis was comparable to LA-C/D esophagitis in terms of acid-suppressive therapy response, esophageal motility, and reflux burden (according to MII-pH parameters), which were significantly different from those of non-RE patients. However, the above parameters did not differ significantly between patients with LA-A esophagitis and non-RE patients. Thus, we further investigated whether adjunctive or supportive evidence defined by Lyon Consensus 2.0 can improve the diagnostic value of LA-A esophagitis. The results indicated that, among patients with LA-A esophagitis, those with >80 reflux episodes per day (LA-A + >80 reflux episodes vs non-RE: 90.0% vs 52.4%, *P *=* *0.044) or hypotensive EGJ (LA-A + hypotensive EGJ vs non-RE: 72.4% vs 52.4%, *P *=* *0.040) had a significantly higher acid-suppressive response rate than non-RE patients (Figure 2).

## Discussion

With changes in life style and the raise in people's concern regarding quality of life when it comes to health issues, GERD has gradually been regarded as one of the most common digestive diseases, whose prevalence is still growing rapidly [24]. GERD can be divided into non-erosive reflux disease, RE (LA-A to LA-D), and Barrett's esophagus according to endoscopy [1]. Recently, Lyon Consensus 2.0 recommended LA-B esophagitis as definitive diagnostic evidence (similarly to LA-C and LA-D) for GERD [18]. Our results demonstrated that LA-B esophagitis was comparable to LA-C/D esophagitis in terms of acid-suppressive therapy response rates, esophageal motility, and reflux burden (according to MII-pH parameters), which were significantly different from those of non-RE patients. These results supported LA-B esophagitis as conclusive diagnostic evidence for GERD. Although LA-A esophagitis differed from non-RE, the differences did not reach statistical significance. The results indicated that LA-A esophagitis was borderline or inconclusive evidence for GERD diagnosis and should be combined with other adjunctive or supportive evidence such as the number of reflux episodes exceeding 80 per day or hypotensive EGJ.

Endoscopy plays an important role in the diagnosis of GERD, especially in countries and regions with a high incidence of gastrointestinal tumors (such as China), where endoscopy has become the preliminary examination for patients with reflux symptoms [5]. However, esophagitis was found in only 30% of treatment-naive patients with reflux symptoms and in <10% of patients who already took acid-suppressive therapy [25, 26]. Furthermore, most of those were low-grade (LA-A or LA-B), especially for Asian countries, where LA-A and LA-B esophagitis accounts for >95% of esophagitis [10]. Esophagitis does not necessarily mean GERD. Mucosal damage can be caused by other factors such as medications, food, immune-related disorders, etc. [27]. Low-grade esophagitis (LA-A or LA-B) can be found in ∼5.0%–7.5% of asymptomatic controls [11–13]. Therefore, for a long time, it has been generally believed that the diagnostic value of different grades of esophagitis for GERD is different. LA-C/D esophagitis has long been accepted as being conclusive evidence for GERD, while LA-A esophagitis alone is considered insufficient to be definitive diagnostic evidence of GERD. As for LA-B esophagitis, controversy arises. Lyon Consensus 1.0 believed that it should be taken as borderline evidence for GERD (similarly to LA-A esophagitis) [14], while ACG clinical guidelines suggested it can be diagnostic of GERD in the presence of typical GERD symptoms and PPI response [4]. In Asia, where most of the countries have a lower reflux burden than Western countries, LA-B esophagitis alone was thought to be enough to diagnose GERD [5, 15]. Recently, Lyon Consensus 2.0 was updated from Lyon Consensus 1.0 to indicate that LA-B/C/D esophagitis should be considered conclusive evidence for GERD. This update is supported by two key studies. In the first study, by Visaggi *et al.* [17], it was found that 100% of patients with LA-B esophagitis showed objective evidence of GERD on MII-pH compared with only 17.6% of those with LA-A esophagitis. Additionally, the response to acid suppressors was similar between patients with LA-B and LA-C esophagitis. The second study, by Rusu *et al.* [16], conducted prolonged 96-hour pH studies with 39 healthy controls and 944 patients. Their analysis showed that the average AET was effective in distinguishing healthy individuals from patients with LA-B/C/D esophagitis, though it did not differentiate between those with grade A esophagitis. Our study confirms for the first time that the recommendation regarding LA-B esophagitis in Lyon Consensus 2.0 is also applicable to an Asian population. The first inclusion of non-esophagitis patients with reflux symptoms was performed to compare the acid-suppression response with esophagitis patients. Also, we verify whether ancillary evidence can improve the rate of acid-suppression response in LA-A esophagitis. Our findings suggest that there are no significant differences in endoscopic findings, esophageal motility, reflux burden, or response to acid suppression between patients with LA-A esophagitis and those without esophagitis. This suggests that LA-A-grade esophagitis alone does not provide a conclusive diagnosis of GERD. The possible reasons for the low specificity of LA-A esophagitis are as follows [28]: (i) it can be caused by other factors (food, drugs, etc.); (ii) the severity of the reflux of these patients is relatively mild; (iii) its correct detection and identification are highly subjective (as only erosion of the esophagus of <5 mm is classified as LA-A). This study evaluated whether the adjunctive and supportive evidence defined by Lyon Consensus 2.0 could improve the acid-suppressive response rate of LA-A esophagitis. Although the sample size for patients had concurrent LA-A esophagitis and the adjunctive or supportive evidence for GERD was small, our study did find that, among patients with LA-A esophagitis, the number of reflux episodes exceeding 80 per day or hypotensive EGJ tended to be a strong indicator of a positive acid-suppression response. Further studies with large sample sizes are still needed to assess whether other evidence can similarly improve the acid-suppressive therapy response in patients with LA-A esophagitis.

All patients who were enrolled in this study had typical reflux symptoms. Do the results of this study apply to asymptomatic patients? Our previous study involving 248 patients evaluated the clinical outcomes of asymptomatic low-grade esophagitis. The results demonstrated that asymptomatic LA-B esophagitis had significantly worse clinical outcomes (mucosal healing rates, the probability of developing symptoms in the future) than asymptomatic LA-A esophagitis [28]. The results suggested that it was inappropriate to consider LA-B and LA-A esophagitis as evidence of the same grade in the diagnosis of GERD. Even in the absence of reflux symptoms, acid suppression should be initiated for LA-B esophagitis.

This study also had several limitations. Firstly, it was retrospective. Secondly, there were only a few patients who had concurrent LA-A esophagitis and adjunctive or supportive evidence for GERD. This prevented us from fully evaluating whether adjunctive or supportive evidence can improve the diagnostic efficacy of LA-A esophagitis for GERD. Lastly, as stated above, the reflux burden between Asian and Western countries varies greatly. This study only included Chinese esophagitis patients. Whether the results of this study are applicable to other countries remains to be further investigated.

## Conclusions

This study demonstrated that LA-B esophagitis can be regarded as conclusive evidence for GERD and initiate acid-suppressive therapy. LA-A esophagitis was unable to establish a definite GERD diagnosis alone. When combined with adjunctive or supportive evidence, the acid-suppressive therapy response rate of LA-A esophagitis improved.

## Authors’ Contributions

J.C., P.D. and S.C. were responsible for acquisition of data, analysis and interpretation of data, and drafting of the manuscript; Q.W. and Y.X. were responsible for study concept and design, analysis of data, and final drafting and approval of the manuscript; Q.Z., N.T., K.S. and F.T. were responsible for acquisition of data and interpretation of data. All authors have read and approved the final version of the manuscript.
